# From Risk Assessment on Site to How to Improve Safety: An Easy “App” to Control Construction Site Conditions

**DOI:** 10.3390/ijerph20053954

**Published:** 2023-02-23

**Authors:** Francisco J. Forteza, José M. Carretero-Gómez, Barbara Estudillo, Albert Sesé

**Affiliations:** 1 Department of Industrial Engineering and Construction, University of the Balearic Islands, Ctra. de Valldemossa, Km. 7.5, 07122 Palma de Mallorca, Spain; 2 Business Economics Department, University of the Balearic Islands, Ctra. de Valldemossa, Km. 7.5, 07122 Palma de Mallorca, Spain; 3 Department of Psychology, University of the Balearic Islands, Ctra. de Valldemossa, Km. 7.5, 07122 Palma de Mallorca, Spain

**Keywords:** safety inspection, digital technologies, App, CONSRAT, construction risk control, organizational site resources

## Abstract

A construction site has specific risks and organizational conditions requiring on-site safety inspections. Paperwork inspections have important limitations that can be overcome by substituting paper records with digital registers and using new information and communication technologies. Although academic literature has provided several tools to carry out on-site safety inspections adopting new technologies, most construction sites are not currently ready to adopt them. This paper covers this need of on-site control by providing an application that uses a simple technology accessible to most construction companies. The main objective and contribution of this paper is to design, develop, and implement a mobile device application (App), named “RisGES”. It is based on the model of risk that grounds the Construction Site Risk Assessment Tool (CONSRAT) and on the related models that connect risk with specific organizational and safety resources. This proposed application is aimed to assess the on-site risk and organizational structure by using new technologies and considering all relevant resources and material safety conditions. The paper includes practical examples of how to use RisGES in real settings. Evidence for the discriminant validity of CONSRAT is also provided. The RisGES tool is at once preventive and predictive since it yields a specific set of criteria for interventions intended to decrease the levels of risks on-site, as well as to detect improvement needs in the site structure and resources for increasing the safety levels.

## 1. Introduction

Most construction safety studies begin by highlighting the high levels of accidents and unsafe conditions [1], which in turn affects their incident rates, being higher than the average of other industries [2,3].

A Safety Management System (SMS) is essential to managing construction safety, and it always includes safety performance measurement and evaluation [4,5]. Safety inspections are crucial to ensure that SMS works as expected [2,3]. It is not enough to evaluate, but it is necessary to establish sufficient control measures to reduce risks [6].

Only a few studies deeply explore this topic despite its importance [5]. The efficiency and limitations of current safety inspections constitute one of the great problems pointed out in the literature [5,7]. Collecting data using the traditional paper-and-pencil procedure is tedious, inefficient, and an error-prone system [5,7,8]. It is difficult to identify and correct safety deficiencies in time, given the large amount of work involved in an inspection, including data processing, and the workplaces’ specific characteristics, such as size. For this reason, new technologies that allow for faster and more efficient decisions should be incorporated [6,9,10].

Many innovative technologies are available since current research has reinforced the proposal for using new technology and information systems to improve safety conditions on-site [11]. Current trends focus on automating data capture and analysis, using digital media on mobile devices to facilitate fieldwork and processing of the results [5,12]. Among the new advances to control conditions in the construction sector, there are virtual reality, augmented reality, mixed reality, real-time monitoring, and design-adapted prevention through Building Information Modeling (BIM) [6,12,13].

According to Tanvi-Newaz et al. [6], these new technologies also entail important limitations such as the cost and complexity of capturing and processing data on-site, or the lack of experts to apply them. These may be the main reasons why these systems are still far from the daily activity in the construction sector.

It should also be noted that the construction industry consists of mostly small and medium enterprises (SMEs). According to EUROSTAT [14], the construction sector in the European Union is made up of 99.4% of small companies employing 74.0% of construction workers. The sector is so fragmented that 93% of the companies can be considered microenterprises, that is, they employ less than 10 people. Other countries have a similar composition, for example, in the United States, 63% are small construction companies [15]. In addition, SMEs report the most accidents, and these kinds of companies are the ones that tend to have fewer resources, specifically, those related to safety management [16]. This lack of management resources leads to a low implementation of management systems in SMEs, but even when they exist, they are possibly highly questionable [17]. 

Owing to the requirements and limitations of the new technologies mentioned by Tanvi-Newaz et al. [6], their use is restricted to large companies with abundant resources and highly qualified technical personnel. Therefore, most of today’s construction companies are left out of this trend, because of the characteristics of the sector. 

Any technology and its implementation must be based on clear and specific messages or instructions; otherwise, any investment in on-site inspection will be inefficient because it is likely that the information will never reach the person(s) who must implement the intervention.

One of the most important indicators involving safety management issues are safety inspections and observations [18]. In consequence, the link between management performance and safety conditions is important to be considered as a leading indicator [19]. Each construction site has its structure and resources that influence physical site conditions, affecting first, the risk level, and finally, increasing the accident rates [20,21]. In a complete on-site inspection, it is necessary to review both the risk conditions and the key organizational factors, such as safety resources, the health and safety plan, crew responsibility, meetings, training, etc. [1,2,20,21,22].

Academic research has provided a few tools to assess on-site risk levels, but they do not take into account the on-site organizational conditions affecting risk and neither provide clear guidelines nor a specific set of criteria for interventions [20,23]. There is “a need for more efficient and effective information management systems for construction” that avoids the inconveniences of paperwork [7].

To fill this gap, this article proposes a new procedure that follows the nature of the existing tools in the literature, by adopting a broad multiperspective approach. An App in line with the one suggested by Lin et al. [11] and Zang et al. [24] is proposed. The App is built upon previous studies concerning site risk assessment [20], the relationship between organizational factors and risk levels [21], and finally, the specific risks that can be affected by on-site organizational and resource level [25]. All these previous studies provide theoretical support for our App and provide empirical evidence for the validation of the procedure.

Summarizing, the main goal of this study is to provide the details of a new and original technological application that is grounded in previous theoretical and empirical studies. The App developed and reported in this paper is adaptable to the special conditions of each site, and it can capture the sites’ structure and the available on-site resources. Using this information as input, the App facilitates the keys for early intervention by generating a proposal for a set of criteria for action to improve both the organizational resources and the on-site risk conditions. Additionally, regarding the description, explanation, and presentation of the application, this paper also reports examples of how to run the application for three different real construction sites.

The paper is structured as follows. The Methods section offers the theoretical basis and the validation process of the App. The Results section reports several practical examples of the application of RisGES, including graphical examples of the App’s output. In the Discussion and Conclusions section, the results and main conclusions are drawn. Finally, in the Appendices are some examples of reports generated by RisGES as a result of on-site inspections.

## 2. Materials and Methods

### 2.1. Problem Statement and Purpose

This study aims to develop a safety management system for construction sites through an App with a user-friendly interface, which adopts the new Information and Communication Technologies (ICT) [1] trends that try to correct the problems of the classic paper-and-pencil procedures, and at the same time, attempts to avoid the complexity and high price of some existing technologies. The key elements for creating this tool are to consider the multiplicity of factors converging from worksite risks and their potential precursors, and to identify the most important safety-related indicators to be assessed.

Based on our previous research, the theoretical App’s core is proposed as a relational structure model composed of on-site risk variables and organizational factors. The App puts into operation in digital format the CONSRAT instrument (Construction Site Risk Assessment Tool) [20], a tool for capturing the specificities of on-site risks and also considers the empirical evidence obtained by two subsequent studies on the relationship between risk indicators and organizational variables [21,25]. The purposes of this relational model are to promote on-site safety inspections and to propose appropriate interventions based on an easy-to-do assessment procedure for both risk indicators and organizational factors and resources. The following two subsections describe the main elements of the relational theoretical model and the empirical evidence obtained after its development and validation.

### 2.2. Site Risk Assessment Model: The CONSRAT Instrument 

CONSRAT is an existent instrument that considers each construction site as a unit of analysis where potential risk synergies emerge when individual risk variables coexist on-site (Forteza et al., 2016). It takes a site risk approach using ten risk variables (RV) to assess main live conditions and ten organizational risk-related variables (OV). Table 1 shows the composition of all risk and organizational variables. The rationale and foundations for all variables are detailed in Forteza et al. [20].

Five of the ten risk variables are considered alarm variables (with an asterisk in Table 1), since they point out severe problems to be prioritized. Each risk and organizational variable is formed as the result of different combinations of a set of site items that each technician must assess. For example, the items related to the RV5 (falls of height) are the height of fall, level of failure, exposure continuation, probability, severity, and intervention required. Risk variables represent continuously changing live site conditions, while the nature of organizational variables is more static. The tool provides a single evaluation of the items related to the organizational variables, and multiple and continuous evaluations of the items related to the risk variables. 

CONSRAT combines different response scales to assess risk indicators. The tool provides a four-level scale ranging from “0” meaning complete compliance level to “3” meaning very poor or noncompliance. So, each level can be assigned a value ranging from 0.00 to 1.00 with equivalent increments of 0.33. A dichotomous scale is available to assess other indicators as both the presence or absence of risk or the adequacy of protective measures. 

Following these general assessment criteria, the items are classified by one of the three levels reported in Table 2. From this, direct intervention criteria are obtained based on whether the item has been in the high range of the scale. Each assessed score of a risk variable is matched with its corresponding intervention level depending on the risk range. Table 2 shows the three scoring levels of the risk variables and the corresponding general intervention criteria that are common to all risk variables. Examining this procedure in greater detail, the App implements specific intervention criteria according to the risk variable outcomes. This assessment procedure aims to provide easy and clear guidance for on-site practitioners, given the nature and peculiarities of the construction sector and the limited command structure at most construction sites, as it is mentioned above. The goal is to simplify the interventions derived from the results of the risk variables assessment. 

After having discussed the assessment site risk variables, the focus is on the assessment of organizational factors that CONSRAT provides. The instrument considers two types of organizational variables; those related to the site complexity and those related to the available resources and means. Similar to the structure of the risk variables, the organizational variables (Table 1) are built with different items having a scale ranging from 0 to 1. For example, the “internal organization structure” (OV6) contains four items: type of contracting, number of companies at the site, level of subcontracting, and number of works. In the case of OVs, this measurement range cannot be divided into specific intervals for pre-established levels of accomplishment. There is no sense in establishing a predefined level for these variables and their range because each site has its specificities regarding complexity and needed resources. In other words, there is no adequate level in terms of organizational variables, but what must be appropriate is the relationship that they maintain with the risk variables for a construction site. The following subsection describes the main evidence found on the relationship between organizational and risk variables using the CONSRAT tool [21,25]

### 2.3. Relationships between Risk and Organizational Variables On-Site

The question up to this point is how to empirically connect safety intervention recommendations about organizational factors that impact the risk variables assessed at a specific site. Two studies using the CONSRAT tool have provided relevant evidence on the connection between the organizational and the risk variables [21,25]. The first one obtained evidence for the relationship between the Site Risk Index (SRI), estimated as the average of the ten RVs, and four organizational clusters measured through the clustering of the ten OVs of the site: F1 (Site complexity), F2 (Firm’s structure resources), F3 (Site structure complexity), and F4 (Safety management resources) [21]. In the second and more recent study, Forteza et al. [25] have proposed an alternative specification of the relational model connecting risk on-site and organizational factors. Based on empirical data, their idea is to identify and clarify the most significant relational paths connecting specific RVs with organizational factors as precursors. This approach decomposed the RSI into its ten RVs and hypothesized the OVs into two broader latent factors representing organizational Complexity (F1) and organizational Resources (F2) using structural equation modeling techniques.

**Figure 1:**
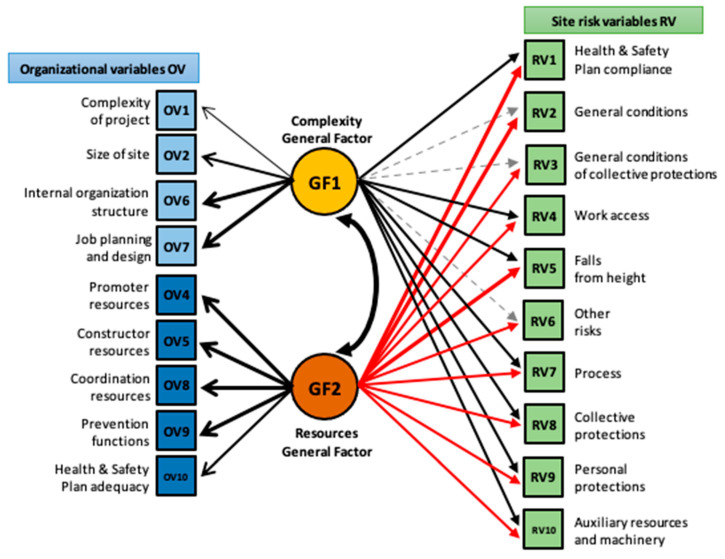
SEM model connections between risk and organizational variables. Modified from Forteza et al. [25].

Figure 1 shows the stronger relationships found among risk and organizational variables using a SEM model [25]. The thickness of the arrows symbolizes the relative magnitude of the effect size of the connections between variables. The black arrows in the model represent positive path coefficients, while the red color indicates a negative relationship. The gray dashed arrows indicate nonsignificant relationships. The model focuses on the most relevant organizational variables and their potential interventions in a complementary way to the safety actions derived from the assessment results of the site risk variables. For example, the RV2 (General conditions on-site), composed of assessing the fence, circulations, order, and tidiness, among others, is strongly influenced by the GF2 (Site resources) with a negative relationship. In this way, in addition to implementing the appropriate interventions to mitigate the specific risks composing RV2, improvements in the organizational variables (OVs) that make up GF2 can also be carried out, which, in turn, preventively may decrease such risks. Consequently, for the GF2-RV2 relationship, it is necessary to inspect what is happening in the behavior of the OVs; for example, with OV9 (Preventive functions of the structure), because perhaps the responsible agents have not assumed them well or at least sufficiently.

In summary, the theoretical and empirical background for the App is the tool CONSRAT [20] and its empirically tested model that posits consistent relationships among on-site risk variables and organizational factors [25].

### 2.4. RisGES App Development

The mobile RisGES App was developed for iOS and Android. Both versions have the same functionalities, and consequently, the internal structure is the same. However, they do not share the same source code because they were developed in a native form in order to optimize the performance and specific characteristics of each platform. The iOS version is developed with Xcode, in Swift + UIKit language. The Android version is developed in Android Studio, in Kotlin language. Both use the same web service as the backend. This was developed in Python + Django + MySQL, run on Linux. Both are common programming languages for mobile applications. Swift is the main language used for developing native applications in iOS, and Kotlin is often used in the case of Android, although it is not the most popular. 

The programming of RisGES was carried out to achieve several purposes. One is the simplicity of data entry and the automatic evaluation of risk and organizational variables. The other is to facilitate the users’ subsequent analysis of the data and its evolution, and provides a guide to intervene at the site. To be able to carry these out, the application implements the following functionalities: definition of the site, realization and consultation of the valuations, analysis of results by means of graphs, and generation of reports. 

To make all of this possible, each of these functionalities was assembled, validating their operation, testing and correcting all failures detected in the process. 

Currently, RisGES is in beta version and has been installed by more than 700 technicians associated with the foundation that has promoted the App. Previously, it was tested at different sites, and has the credit of validity checks for CONSRAT matrixes using a sample of more than 1000 construction sites [20,21].

In the next section, this study offers all the details for the newly created App and the process required for using it. In addition, there are three different examples for using RisGES. These examples were chosen because of their different levels of risks and resources, site topologies, and phases of work. This is intended to show different applications for various examples together with its operation. The tool was administered by the same technician, one of the authors of this study, accompanied by the person in charge of each site and using the App to answer each question. 

## 3. Results

In this section, three examples of how to use RisGES in real settings are provided, and the results from additional tests for the discriminant validity of CONSRAT that were conducted. 

### 3.1. RisGES Process

Using RisGES is a five-step process. In the first step, the technician must complete the site register including site identification and characterization. There are about 17 questions that include promoter and constructor characterization (see Appendix A for captures of the screen for this phase). This step needs to be completed just only once per site. The second step must be completed every time an on-site inspection is carried out. It collects data about the stage of the work and risk factors, requiring different evaluations for several aspects such as general conditions, access to the workplace, falls from height, other risks, process, collective protections, anti-fall protections, auxiliary resources, and machinery. It involves 78 questions in which the technician just needs to select a predefined option in most cases. There are 36 compulsory questions to answer; in most cases, the user can add new options (see Appendix A for captures of the screen for this phase of characterization). The third step consists in visualizing the main two graphics of the App that characterize risk and organizational variables, as it can be seen in Figure 3. The fourth step offers the possibility of generating different types of graphics, for example, showing the evolution of a single site through time, and a comparison of different sites (see Appendix B to check all types of graphics that RisGES generates). Finally, the fifth step gives a complete feedback report with all the information on how to intervene on-site (see Figure 4 for a screen capture of this step and Appendix C to check the complete site inspection report). 

After steps three and five, the RisGES App gives the possibility to obtain a report of the results. The first report is simply the graphic of the variables (Figure 3). The second report comprises overall information but includes specific guidelines for on-site interventions. 

### 3.2. Use of RisGES: Construction Site Examples

**Figure 2:**
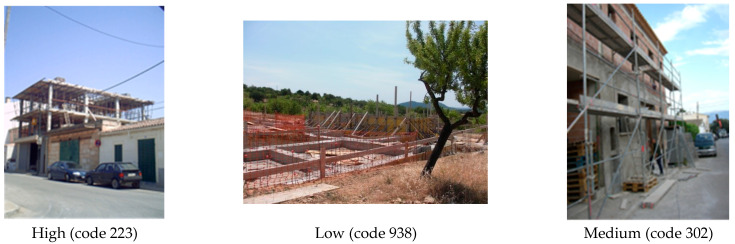
Pictures of the three different sites classified by their level of risk. From left to right: high (code 223), low (code 938), and medium (code 302). Source: from the authors’ archive.

**Figure 3:**
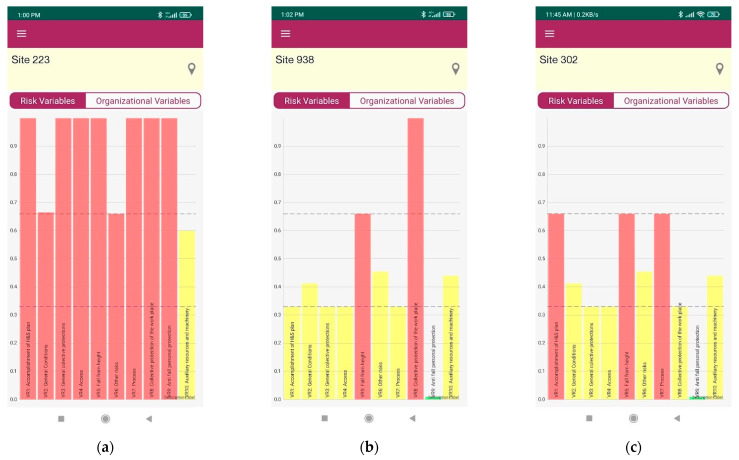

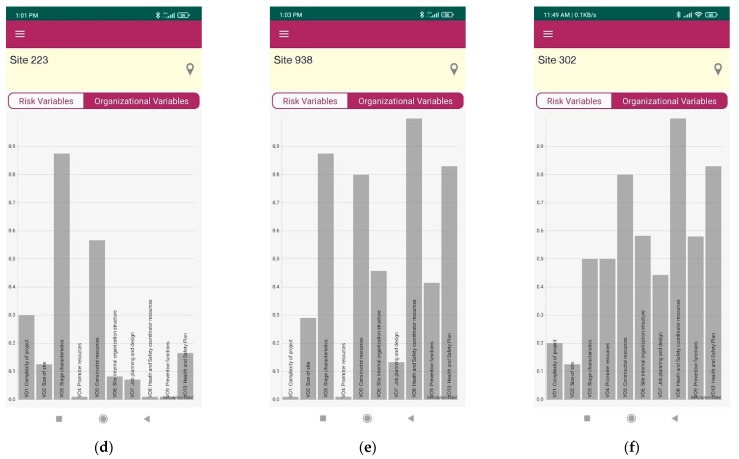
Examples of graphs representing the measurement levels for the risk (**a**–**c**) and organizational variables (**d**–**f**). From left to right: site 223, site 938, and site 302. Source: App screenshots.

Table 3 shows the different risk and organizational variables obtained on three sites with different levels of risk: one with a high level of risk (site code 223), another with a low level of risk (site code 938), and finally, the last site with a medium level of risk (site code 302). This is the information obtained after the mentioned third step that corresponds with the graphics in Figure 3. Numerical results in Table 3 are obtained after valuing each item of the second step (78 questions) mentioned above that the technician must complete according to CONSRAT methodology [20] (pp. 346–353). In Appendix A, the visual process to accomplish all these steps using the App can be seen. As shown, the numerical results of Table 3 are consistent with the pictures shown in Figure 2 and Figure 3. 

### 3.3. Risk-Level Assessment

As shown in Figure 3, Graphics of risk variables, these graphics provide a fast and intuitive image of the assessed risk level on-site at a given moment. For example, the site with code 223 obtains the highest level of risk. This site has 9 of the 10 risk variables at critical levels according to the Table 2 classification, and just 1 risk variable at an acceptable level (VR10). On the contrary, the site with code 938 only presents two values at a critical level; they are the variables RV5 and RV8, which correspond, respectively, to the risks of “Falling from height” and “Collective protection” (Table 2). Additionally, in the site with code 938, one risk variable shows good behavior (VR9) while the other seven risk variables show behaviors within the acceptable interval. Obviously, the fact that this site has a lower general level of risk than the previous one does not mean that it does not require any intervention. In fact, it needs it, since the deficiencies affect variables directly involved with the most important risks in construction, such as “Falling from height”. As shown in Table 1, both variables (RV5 and RV8) are classified as “Alarm variables”, which means that they need special control and vigilance, because they involve risks potentially causing accidents with worse consequences. Regarding the site with code 302, it presents medium levels of risk, compared to the two previous ones, since it has three risk variables at critical levels, one risk variable at a good level and the other variables at an acceptable level. Similar to site 938, risk variables 1, 5, and 7 require special attention. These variables correspond, respectively, to “H&S plan compliance”, “Falls from height”, and “Process”, owing to their critical level. Furthermore, as indicated in Table 1, two of them are classified as alarm variables, RV1 and RV5, so they require special attention, owing to the risk they represent. 

### 3.4. Organizational Variables Assessment

The other set of graphs (see Figure 3, Graphics of organizational variables) facilitate the evaluation of the site corresponding to all variables that conform to the organizational factors of resources and complexity. In this case, the application does not classify levels of these organizational variables, but uses them to be associated with the observed level of the risk variables, providing recommendations to improve on-site safety conditions by taking actions on organizational complexity and resources.

For example, the graphic showing site 223’s organizational variables (see Figure 3, Graphics of organizational variables) indicates a low level of complexity (OV1, OV2, OV6, OV7) all of them below 0.3 on a scale of 0–1. The problem is that the resource variables also show very low levels (OV4, OV5, OV8, OV9, OV10). Except for OV5, which has a value of 0.56, the others have very low values, such as OV10 with a level of 0.16 and the others with levels of 0 resources (OV4, OV8, OV9). These values mean that in this case, the site presents little constructive complexity, but with very poor levels of organizational resources, which are correlated with the identified high levels of risk. Regarding site 938, it presents a different graph of organizational factors (see Figure 3, Graphics of organizational variables). Similar to previous site 223, site 938 shows a complexity factor composed by organizational variables with low observed values (OV1, OV2, OV6, OV7), three below 0.3 and one (OV6) with a value of 0.46 on a scale of 0–1. However, unlike site 223, site 938 shows more important levels of resources (OV4, OV5, OV8, OV9, OV10). Only the OV4 variable shows a value of 0, while the other organizational variables range from 0.58 (OV9) to 1 (OV8). 

### 3.5. Other Utilities of RisGES: Graphics Comparison

To complete the information on these two basic graphics, the App facilitates up to six more graphics that can be seen in Appendix B. The first graphic, with the bottom label “G3:RV 2S/1P”, shows the comparison between the risk variables of two sites and one construction phase, in this example, the flat roof, where the comparison of site 395 with another site can be seen. The second graphic in Appendix B (that with the bottom label “G4:RV 1S/1P/DB”) reports the comparison between the risk variables of one site with the database of the user’s account of the App. In the example illustrated, we can see site 938 in its phase of “foundations and structure”. The gray bars reflect the observed levels of each risk variable, while the green, blue, and red lines report the lowest, average, and maximum levels in the database, respectively. The third graphic, shown in first row of Appendix B (graph with bottom label “G5:RV 1S/1P”), indicates the comparison between the risk variables of one site and several work phases. In this example, we can see site 395 and the phases of “excavations”, “foundations and structure”, “brickwork”, and, finally, “flat roof”. This is a graphical way to check the evolution of risk on-site along several works. The next three graphics in Appendix B use the risk on-site index (SRI) to analyze the evolution of risk on-site as the average of all risk variables. Thus, the graph with the bottom label “G6:SRI 1S/1P” shows the evolution of risk on-site (SRI) along several works phases. In this example, for site 395, there is a clear increase in risk along the four construction phases. The graph with the bottom label “G7:SRI 1S/1P” illustrates the SRI of several sites and several work phases are very similar to the previous one, but in this case with two sites. Finally, the graph with the bottom label “G8:SRI 1S/1P/DB” is a comparison between the evolution of SRI on-site for different phases and the database of the App technician. In this case, the site (in blue) is situated near the average (in gray). 

### 3.6. Final Report

**Figure 4:**
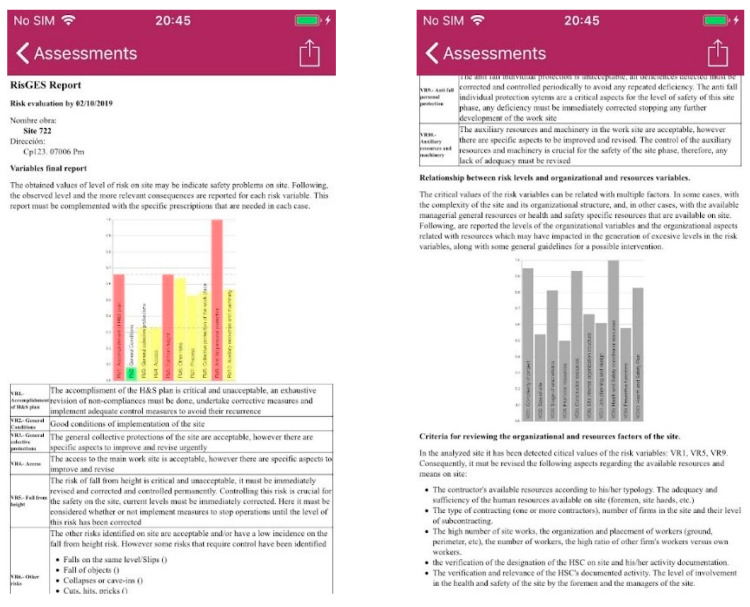
Examples of the screen capture of site inspection and recommendation reports. Source: App screenshots. (See Appendix C to read the complete sample reports).

Finally, the RisGES App provides a report that comprises the full information for initiating the intervention on-site. Figure 4 shows captures of the screen with the report that the user can immediately check at the end of the assessment (see the full version of the report in Appendix C, for the case of site 223 and its “structure” phase). The report provided by the App is in HTLM format, which allows for easy editing and sharing by using e-mail, WhatsApp, among others. In this example, an excerpt of the default report yielded by RisGES is reproduced, which can be edited by the technician in order to personalize each site according to its specificities.

As mentioned above, the App provides an inspection report that the technician can complete and personalize. This report materializes the recommendations for improvement at the risk and organizational levels following the research obtained in Forteza et al. [25]. The risk-level report for site 223 is shown in Appendix C, which is the one that attained the highest risk-level; it corresponds to an assessment during the phase of “structure”. This site consists of a small, new single-family house without special environmental conditions distributed on three different levels (ground floor and two upper floors). The main work stage corresponding to the inspection is the structure. The promoter is professional, the main constructor supervising the site is a self-employed constructor, he or she is the contractor (direct contract relationship with promoter), and the business owner is the leader at the site. There are two contractors on-site, the one mentioned, who is self-employed, and another there who is not subcontracting. The construction site presents just one main work; there are three workers on the perimeter floor, at the third level, about 9 m (29.5 feet) above street level. There is no documentation to demonstrate health and safety coordination; therefore, there is no documented work from them. The health and safety plan is on-site, and its provisions are unknown for the interlocutors on-site. All this information is aggregated to conform to the mentioned organizational variables. 

As shown in Appendix C, the report begins with the commented graphic of risk variables. This is an extreme case where nine of the ten risk variables obtained critical values, additionally, seven out of the nine variables obtained maximum values of 1. The comments of the report reflect the bad results obtained in the inspection and the main recommendations that can be implemented in each case. 

The critical risk values detected in most of risk variables (RV) required an intervention on the material conditions on-site and on its resources, according to their level of complexity. 

In addition, in Appendix C, there is also a comment regarding the observed behavior for each risk variable. For example, VR5, “Fall from height”, which obtained a level of risk of 1, has an extreme comment to draw immediate attention to the elements that affect this variable:

“The risk of fall from height is critical and unacceptable, it must be immediately revised and corrected and controlled permanently. Controlling this risk is crucial for the safety on-site, current levels must be immediately corrected. Here it must be considered whether implement measures to stop operations until the level of this risk has been corrected”.

The default model of the report generated by the App can be edited and personalized by adding, for example, mention of a specific protection problem or work development. In a similar way, the report provides specific comments for each of the risk variables in accordance with their observed behavior. Those comments are the basic criteria for determining the needed interventions.

Next, the report of the inspection provides some recommendations to be addressed concerning the organizational and resource variables (see Appendix C) based on the levels of risk detected and the relationship between risk levels and organizational and resources variables established by Forteza et al. [25]. In the case of site 234, considering the observed high levels of risks and poor levels of resources, the recommendations on resources and organizational variables are also important. According to the theoretical model of connections between risk and organizational variables (see Figure 1), the report of the inspection highlights again which risk variables show worse behavior and their most salient connections with the organization variables. In site 234, the recommendations about complexity and resources affect mainly the project complexity and environment, the constructor resources, the type of contracting, the organization and placement of workers (ground, perimeter, etc.), and site coordination and resources, including adequacy of the health and safety plan.

As a result of the site report, the main interventions at the level of organizational complexity and resources could be the following:

Complexity: The high levels of risk in general and specifically the risk of falling from height and the one related to auxiliary means and machinery can be related to the complexity of the site. It is mainly related with a complexity derived from the planning and design of the work and internal organizational structure. This is materialized with the type of contracting (several small contractors) and affects the location of the workers in the perimeter of slabs without protection. 

Resources: The high levels of risk detected on-site, in particular the risks of compliance with the safety plan, general conditions, fall from height, and auxiliary means, may be related to the lack of resources on-site. Specifically, the resources that can be most affected are the promoter’s resources (support and own resources for site safety) and the builder’s resources (available resources according to the type of constructor and the adequacy and proficiency of the person in charge of the site), the coordination resources (designation and actions of the health and safety coordinator, documented activity), preventive functions (involvement in health and safety by the managers and supervisors at the site), and adequacy of the safety plan at the site (presence and adequacy of contents). 

As it can be seen, the graphics and reports focus globally on the interventions at the construction sites and facilities, and the main ways to implement the recommended intervention.

## 4. Discussion and Conclusions

The main objective of this research is to design, develop, and implement an application for mobile devices that help practitioners to conduct a solid assessment of the risk at construction sites and identify broad areas where the health and safety responsibilities must address interventions to reduce the level of risk. The application, named RisGES, is built upon the theoretical model by Forteza et al. [25] that identifies which and how organizational factors, such as the level of resources and degree of complexity at the site, are associated with the specific risks at sites which have been identified as the major causes of injuries and fatalities in the construction sector. Therefore, this paper is an attempt to transfer academic knowledge into professional activity and make it accessible to practitioners and other stakeholders interested in reducing accident rates at construction sites by assessing and controlling risks.

As it has been reviewed, the basis for the theoretical model by Forteza et al. [25] is to identify which are the significant interaction paths between organizational aspects and the most salient risks at construction sites. These findings and the contributions in [20,21] are all that were needed to develop and construct the RisGES App, which is the main goal of this paper. The application RisGES represents a technological innovation that is grounded on theoretical models of risk and measurement models previously validated and published. RisGES allows for conducting systematic and regular assessment of sites, storage, and sharing information; detect in advance where safety risks are higher; and propose more appropriate managerial corrective actions always based on previous theoretical models and empirical evidence on path analyses. In other words, RisGES is a knowledge transference product that is intended to improve the management of H&S issues in real settings.

The RisGES App shares similar advantages with other computerized systems designed to conduct safety inspections, for example, the Smart Inspect created by Rey et al. [12]. First, the use of the application increases the transparency of the safety conditions based on an assessment of the most relevant risks at construction sites. With the graphics described previously (Figure 3, Graphics of risk variables), any user of the App can see briefly which are the most relevant risks on-site. Second, RisGES improves teamwork and simplifies the transmission of information. Compared to manual assessments, the use of digital and computerized systems can solve the inefficiencies in sharing and transmitting information between different teams. Since the assessments of risk and organizational variables on-site in a specific moment of time are stored in the application, any worker in the safety team can always log-in and obtain access to the whole database. Moreover, access to the App is not necessarily restricted only to the safety staff and, potentially, all supervisors, project and company managers, and other stakeholders can receive feedback from the tool in order to analyze safety problems based on actual data. In fact, one potential positive consequence if managers use RisGES for evidence-based decision-making is that their involvement and commitment to safety issues will increase. Third, RisGES can also reduce the assessment time and facilitate the frequency of the assessments. This is especially relevant as one of the improvements in H&S processes in construction that Ramos-Hurtado [13] has stated is achieving a good balance between time-sensitive goals of construction projects and safety issues at construction sites. Less time-consuming risk assessment processes will cope better with stringent deadlines that push to prioritize time and fast completion of tasks over safety and risk avoidance. 

One potential and very interesting utility of RisGES for construction sites is that its value is higher when the database of assessments is larger. It is true, that any isolated assessment and its corresponding report has value, it can also serve as a catalyst for corrective actions. However, the larger the number of evaluations of a particular work and the more works stored in the database, the greater the potential of RisGES. This is due to the utilities described in Appendix B of this manuscript, where it can be seen, among others, the possibility of tracking the different measurements of the risk level for a construction site, for several construction sites, or for the whole database stored in the App. Thus, typical and predominant small- and medium-sized companies in the construction sector, with limited resources and means, can easily use the database collected by RisGES. In this way, it will be easier and more effective for these companies to take a small step in prevention management, taking advantage of the data science provided by RisGES for decision-making, having access to resources that, usually, are only available to large companies having the means to process and use them.

This paper has shown that the use of RisGES along with other digital and computerized technology can improve the efficiency of safety assessment and risk management for construction sites. In this sense, it represents a relevant contribution in the direction pointed out by Rey et al. [12] since it can help to overcome the difficulty of analyzing extensive databases to elaborate safety reports.

Another benefit of RisGES in the field of research is related to the potential of the accumulated data. As the use of RisGES grows and the App gains popularity in the construction industry, a huge database can be built with the cooperation of the sector. This database can be a good first step to developing sound studies of the precursors of accidents in construction sites. The usefulness of such a research line is clear, not only for a company where RisGES is systematically used at all of its sites, but also for researchers comparing the effective H&S management between different construction companies. As Tanvi-Newaz [6] stated, the incorporation of new technology has moved the reactive approach of risk and hazards management to a more proactive one, allowing the real-time monitoring of all activities in the different phases of construction and triggering effective alarms when the risks are high. RisGES represents a first step in this direction, and it can also be useful for building models to predict risks and thereby reduce accidents at construction sites. Tanvi-Newaz [6] (p. 12) concluded that “there is no a one-size-fits-all technology to entirely address… risks, but a combination of risk prediction, prevention, and mitigation technologies should be cohesively applied to guarantee satisfactory safety performance outcomes”. RisGES can have a valuable role in this combination. One evidence that RisGES has been considered as an interesting tool in the construction industry is that a foundation of the sector was involved in the programming and interface design of the application for mobile devices. The foundation currently provides RisGES free of charge to all of its associated members and offers it to other professionals for a fee.

Despite the benefits of the RisGES App, several improvements need to be undertaken to establish future empirical studies. At a theoretical level, the models of risk assessment must incorporate aspects of safety climate, as it is a factor that undoubtedly can increase risk and can be affected by managerial actions. A more comprehensive model of risk assessment is needed to incorporate safety climate issues; once proposed and validated, the RisGES App must incorporate the evidence found to account for the role of safety culture at construction sites, assess its risk impact, and identify the correct improvement actions at a managerial level. Another action to be taken in the future is to analyze in depth the user’s experience with RisGES and obtain valuable feedback from professionals, which is needed to refine and improve the application as needed.

This study shows how the RisGES App has been designed upon theoretical and empirical models that served to assess the risk at construction sites, on one hand, and the relationships of on-site risk factors with organizational factors, on the other hand. RisGES includes all the insights from theoretical and empirical models and, taking advantage of ICT, allows any technician or competent practitioner to undertake an easy evaluation of the risk on-site and provide a clear focus toward critical improvement actions that need to be performed. The RisGES App can be taken as an instrument to bridge the gap between the management of safety (preventive policies) and the management of organizations (endowment strategy). 

Although the RisGES App is currently under testing and refining, we believe it is a good, easy, and attractive tool to enrich safety management systems. Summarizing, with RisGES, practitioners have: (a) appropriate information to rapidly correct on-site safety problems; (b) a more accurate understanding of the safety conditions on-site; (c) a database of risk on-site and linked organizational factors useful for evidence-based decision-making; and (d) reinforcement of the relationships among different stakeholders related to health and safety management. Therefore, RisGES can be considered as a tool that helps to deploy strategies for effective health and safety management and, as a consequence, helps to reduce the extremely high accident rates in the construction industry.

## Figures and Tables

**Table 1:** The risk and organizational variables that comprise CONSRAT.

Risk Variables	Organizational Variables	
RV1. H&S plan compliance *	OV1. Complexity of project	
RV2. General conditions of the site	OV2. Size of site	
RV3. General conditions of collective protection *	OV3. Stage characteristics	
RV4. Work access	OV4. Promoter resources	
RV5. Falls from height *	OV5. Constructor resources	
RV6. Other risks	OV6. Internal organization structure	
RV7. Process	OV7. Job planning and design	
RV8. Collective protections *	OV8. Coordination resources	
RV9. Personal protection *	OV9. Preventive functions	
RV10. Auxiliary resources and machinery	OV10. H&S plan adequacy	

* Alarm variables.

**Table 2:** Intervals of risk variables and intervention criteria.

Scoring Interval	Intervention Criteria
0.00–0.32	Good behavior of the variable; no intervention is needed.
0.33–0.65	The behavior of the variable is acceptable; no critical improvements are necessary
0.66–1.00	Critical and unacceptable behavior of the variable; immediate intervention is mandatory and posterior permanent control.

**Table 3:** Risk and organizational variables for three different construction sites selected by risk level.

**Risk Variables**	**RV1**	**RV2**	**RV3**	**RV4**	**RV5**	**RV6**	**RV7**	**RV8**	**RV9**	**RV10**
Site 223. High	1	0.66	1	1	1	0.66	1	1	1	0.6
Site 938. Low	0.33	0.42	0.33	0.33	0.66	0.45	0.33	1	0	0.44
Site 302. Medium	0.66	0.42	0.32	0.32	0.66	0.45	0.66	0.32	0	0.43
**Org. variables**	**OV1**	**OV2**	**OV3**	**OV4**	**OV5**	**OV6**	**OV7**	**0V8**	**OV9**	**OV10**
Site 223.	0.3	0.12	0.87	0	0.56	0.08	0.077	0	0	0.16
Site 938.	0	0.28	0.87	0	0.8	0.46	0.13	1	0.43	0.82
Site 302.	0.2	0.12	0.5	0.5	0.8	0.58	0.45	1	0.58	0.82

**Figure A1:**
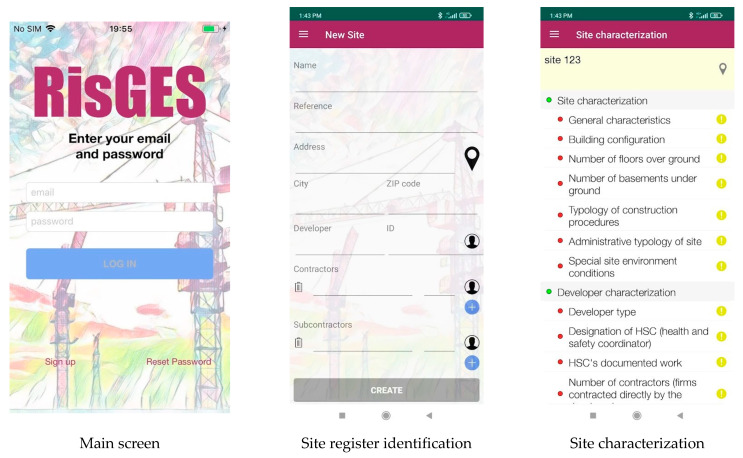
General operation of the application RisGES. Source: App screenshots.

**Figure A2:**
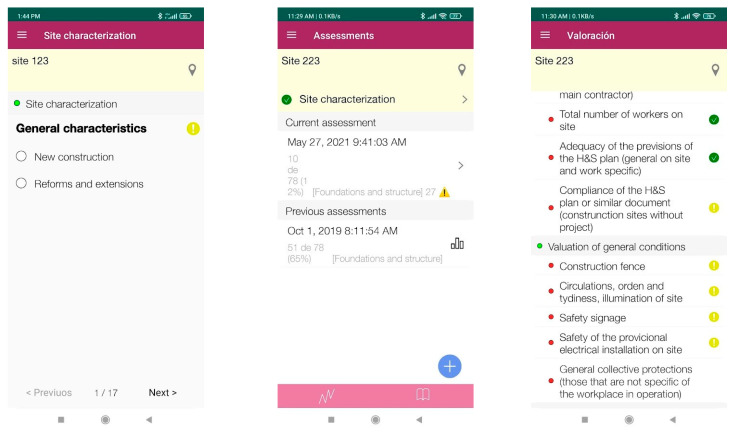
Questions for site characterization. Finalization of characterization and continuing assessment. Source: App screenshots.

**Figure A3:**
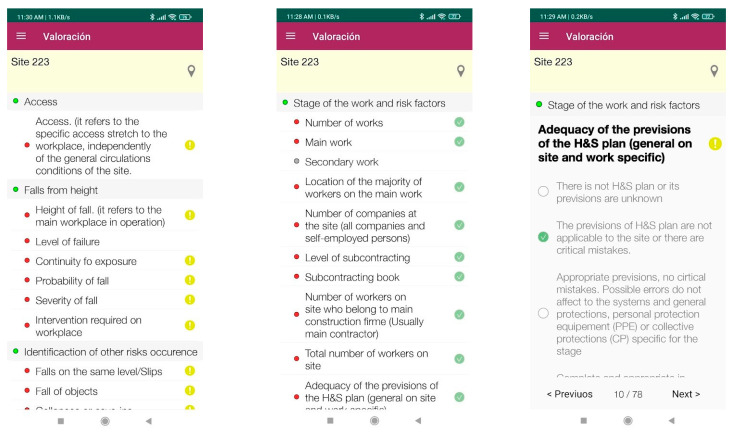

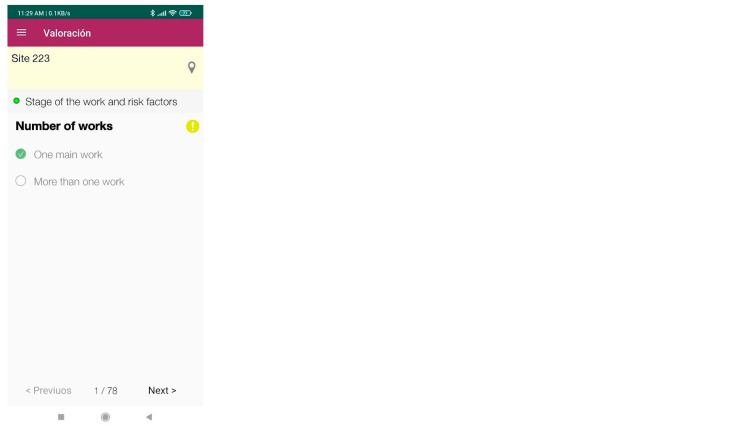
Different screens showing site assessment, general menu, and specific questions with possible answers. Source: App screenshots.

**Figure A4:**
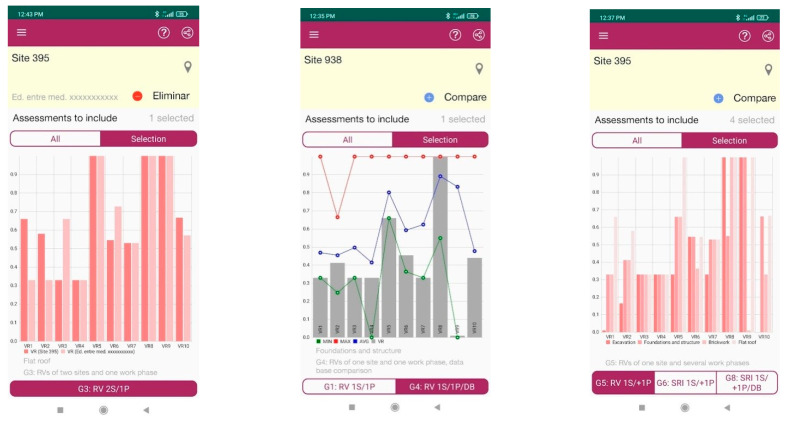

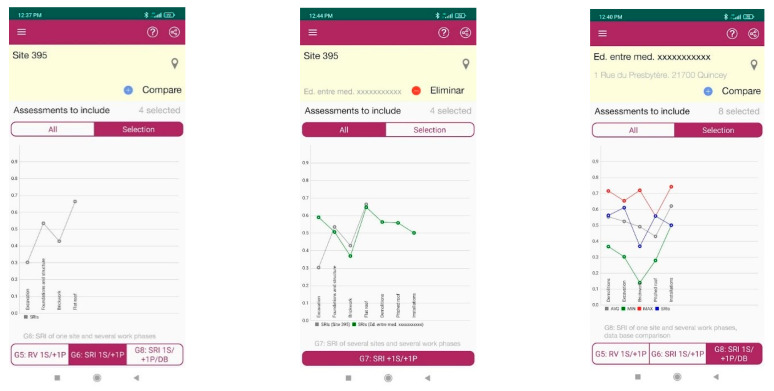
Graphs for control and monitoring of the level of risk that facilitates the application. Source: App screenshots.

**Figure A5:**
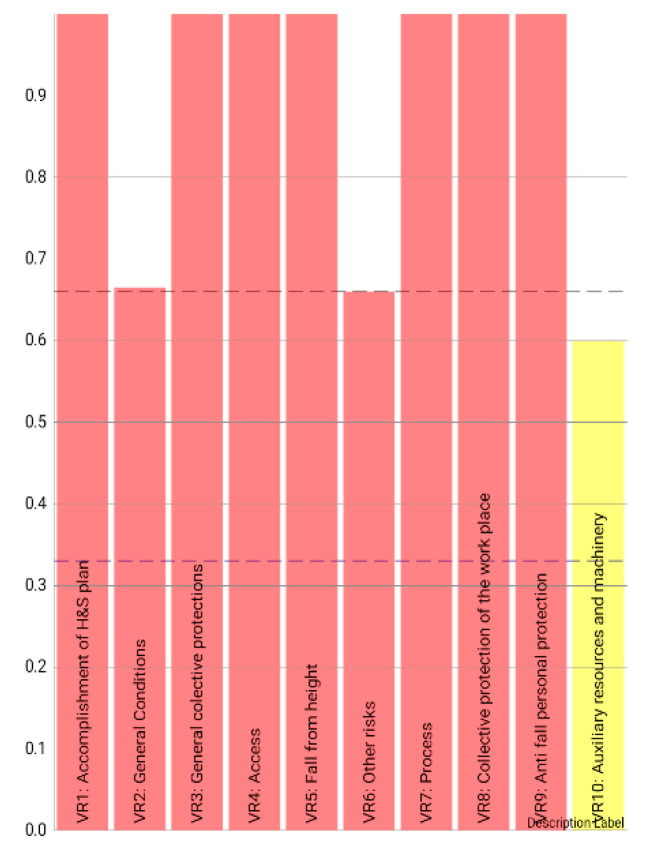
Relationship among risk levels and organizational and resources variables.

**Figure A6:**
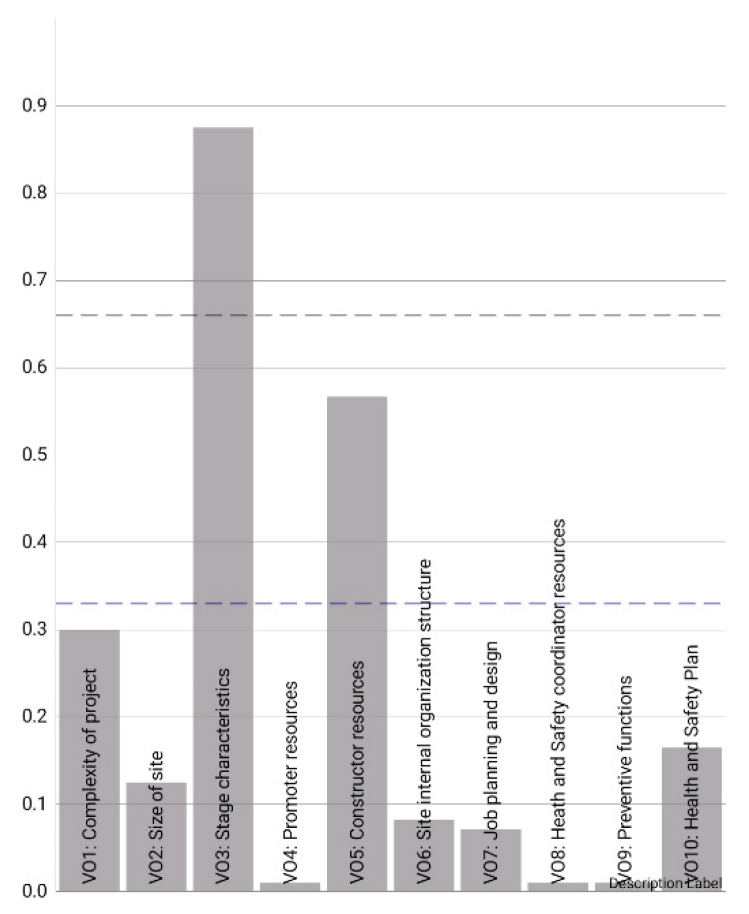
Criteria for reviewing the organizational and resources factors for the site.

## Data Availability

Data are provided upon request to the corresponding author.

## Appendix C

Reporting model with general recommendations that facilitates the application to document the site inspection. Source: App provided report.

RisGES Report

Risk evaluation by 1 October 2021

Site name: Site 223

Address: 234 Pedestrian Route, Palma

Variables final report

The obtained values of level of risk on-site may indicate safety problems on-site. The following shows the observed level and the more relevant consequences that are reported for each risk variable. This report must be complemented with the specific prescriptions that are needed in each case.

**Risk Variable**	**Description**
**VR1. Accomplishment of H&S plan**	The accomplishment of the H&S plan is critical and unacceptable; an exhaustive revision of noncompliances must be performed, undertake corrective measures, and implement adequate control measures to avoid their recurrence.
**VR2. General Conditions**	The conditions of implementation of the site are critical and unacceptable, immediate measures must be undertaken to correct deficiencies and to control periodically.
**VR3. General collective protections**	The general collective protections at the site are unacceptable; they must be immediately revised and corrected, reorganizing the periodical control systems. This is an aspect of radical importance for the safety at the site; if this extends through time, there can be severe consequences. It must be considered whether or not to implement measures to stop operations until the level of this risk has been corrected.
**VR4. Access**	The access to the main work site is unacceptable; it must be immediately revised and corrected and controlled periodically. The access to the work site, more when a risk of fall from height coexists, can be a crucial aspect for the safety of the site.
**VR5. Fall from height**	The risk of a fall from height is critical and unacceptable; it must be immediately revised and corrected and controlled permanently. Controlling this risk is crucial for the safety at the site; current levels must be immediately corrected. It must be considered whether or not to implement measures to stop operations until the level of this risk has been corrected.
**VR6. Other risks**	The other risks identified on-site are unacceptable and/or have a huge incidence on the fall from height risk. The following identified risks must be corrected and periodically controlled: - Falls on the same level/slips; - Fall of objects; - Collapses or cave-ins; - Cuts, hits, pricks; - Hit by a vehicle, crushing, entrapments; - Overextension.
**VR7. Process**	The process is not adequate and/or it presents major deviances. The process must be redefined and/or the control means to guarantee its accomplishment without deviances.
**VR8. Collective protection of the workplace**	The collective protections at the work site are unacceptable; all deficiencies detected must be corrected and controlled periodically to avoid any repeated deficiency in collective protections. CP is a crucial aspect for the level of safety for this phase of the site, any deficiency must be immediately corrected, stopping any further development at the work site.
**VR9. Anti-fall personal protection**	The anti-fall individual protection is unacceptable; all deficiencies detected must be corrected and controlled periodically to avoid any repeated deficiency. The anti-fall individual protection systems are a critical aspect for the level of safety for this site phase; any deficiency must be immediately corrected stopping any further development of the work site.
**VR10. Auxiliary resources and machinery**	The auxiliary resources and machinery at the work site are acceptable; however, there are specific aspects to be improved and revised. The control of the auxiliary resources and machinery is crucial for the safety of the site phase; therefore, any lack of adequacy must be revised.

The critical values of the risk variables can be related to multiple factors; in some cases, with the complexity of the site and its organizational structure, and, in other cases, with the available managerial general resources or health and safety specific resources that are available on-site. Following are the reported levels of organizational variables and organizational aspects related to resources that may have affected the generation of excessive levels in the risk variables, along with some general guidelines for possible intervention.

At the analyzed site, critical values for the risk variables have been detected: VR1, VR2, VR3, VR4, VR5, VR6, VR7, VR8, and VR9. Consequently, the following aspects must be revised regarding the available resources and means on-site:

The complexity of the project, configuration, environment, and/or the size of the site.

The constructor’s available resources according to his/her typology. The adequacy and sufficiency of the human resources available on-site (supervisors, site leaders, etc.).

The type of contracting (one or more contractors), number of firms at the site, and their level of subcontracting.

The high number of site works, the organization and placement of workers (ground, perimeter, etc.), the number of workers, the high ratio of other firm’s workers versus own workers.

The verification of the designation of the HSC on-site and his/her activity documentation.

The verification and relevance of the HSC’s documented activity. The level of involvement in the health and safety of the site by the supervisors and the managers of the site.

The presence on-site, content adequacy, and provision of the HSP.
